# The influence of phthalocyanine aggregation in complexes with CdSe/ZnS quantum dots on the photophysical properties of the complexes

**DOI:** 10.3762/bjnano.7.94

**Published:** 2016-07-13

**Authors:** Irina V Martynenko, Anna O Orlova, Vladimir G Maslov, Anatoly V Fedorov, Kevin Berwick, Alexander V Baranov

**Affiliations:** 1 Department of optical physics and modern natural science, ITMO University, 197101 Saint Petersburg, Russia; 2 Department of Electronic and Communications Engineering, Dublin Institute of Technology, Dublin 8, Ireland

**Keywords:** aggregation, Förster resonance energy transfer, photosensitizer, semiconductor nanocrystals, tetrapyrroles

## Abstract

The formation of nonluminescent aggregates of aluminium sulfonated phthalocyanine in complexes with CdSe/ZnS quantum dots causes a decrease of the intracomplex energy transfer efficiency with increasing phthalocyanine concentration. This was confirmed by steady-state absorption and photoluminescent spectroscopy. A corresponding physical model was developed that describes well the experimental data. The results can be used at designing of QD/molecule systems with the desired spatial arrangement for photodynamic therapy.

## Introduction

Semiconductor quantum dots (QDs) and their complexes with organic molecules have been a subject of extensive research during the last couple of decades. In particular, complexes of QDs and tetrapyrrole molecules were of great interest due to their diverse application in many fields ranging from latest third generation solar cells [1–3] to photodynamic therapy (PDT) [4–10]. Currently, practically all PDT drugs are based on tetrapyrrole molecules. In the PDT process, photoexcited tetrapyrrole molecules undergo intersystem crossing from a singlet state to a triplet state. Energy is then transferred from the triplet state to the surrounding oxygen molecules. This energy transfer converts oxygen to the extremely reactive singlet oxygen, which can destroy diseased cells [11].

It is proposed to use QDs as energy donors, providing “indirect excitation” of tetrapyrrole molecules through Förster resonance energy transfer (FRET), increasing the generation of singlet oxygen by tetrapyrrole molecules in complexes with QDs. Colloidal QDs are particularly suited to the role of energy donor in QD/tetrapyrrole complexes because of their unique optical properties. QDs exhibit an extremely high extinction over a broad spectral range and a high quantum yield (QY) of photoluminescence (PL) [12–14]. In addition, the emission properties of QDs can be tuned through the size of the QD. Thus, the conditions necessary for FRET to occur in QD–tetrapyrrole donor–acceptor pairs can be easily fulfilled.

To date, several studies have demonstrated photoexcitation energy transfer in a variety of QD–tetrapyrrole systems by FRET, with efficiencies close to the theoretical limit for the donor–acceptor pair under consideration [15–17]. However, in many other QD–tetrapyrrole systems real FRET efficiency was significantly lower than was predicted by evaluation of donor–acceptor distance and spectral overlap integral between donor emission and acceptor absorption bands [9,18–21]. This statement was recently supported by the Nyokong group [22] where FRET efficiencies up to 93% were predicted in nanocomposites based on glutathione-capped CdTe/CdS/ZnS QDs covalently linked with aluminium sulfonated phthalocyanine. However, analysis of donor PL quenching and acceptor PL enhancement, which are experimental manifestations of FRET, revealed an unexpectedly weak enhancement of phthalocyanine emission with a simultaneous large quenching of the QD emission, which means a low FRET efficiency. The authors attribute this effect to the presence of nonradiative processes competing with FRET, which deactivate the excited state of the QD. Competing nonradiative processes in these systems usually imply a photoinduced electron transfer or formation of new local surface trap states in the QD induced by the bound acceptor molecule [18,23]. It should be noted that electron transfer in QD–tetrapyrrole complexes is not confirmed by experiment to date. The second mechanism can be only possible in complexes with direct attachment of acceptor molecules to the QD surface, i.e., adjacent capping ligand molecules are replaced with organic dye molecules [24–25]. Therefore, a physical mechanism that implies a low FRET efficiency in QD–tetrapyrrole complexes is still under debate.

In our previous studies it was observed for the first time that an increase in the number of tetrapyrrole molecules in complex with QDs resulted in a significant reduction of the intracomplex FRET efficiency and of the QY of PL of tetrapyrrole molecules [26]. In nonconjugated complexes of cysteamine-capped QDs and chlorin e6 additional channels of nonradiative energy dissipation in QDs and/or in chlorin e6 took place when the relative chlorin e6 concentration in the mixture was increased [19,27].

Aggregation of acceptor molecules in complexes may be the reason for the decrease of the tetrapyrrole photoluminescence intensity in complexes and this may explain the observed concentration dependence of photophysical properties of the complexes. It is well-known that the aggregation of acceptor molecules can dramatically reduce the functionality of the complexes [19]. To our knowledge, there are only a few papers [28–29] devoted to the investigation of how the spatial arrangement of acceptor molecules –in complexes of QD donors with several acceptors– influences the photophysical properties of the complexes. Therefore, this problem important for PDT application needs to be clarified.

Water-soluble sulfonated phthalocyanine derivatives, especially aluminium and zinc complexes, are well-understood sensitizers for PDT and, at the same time, they easily form nonluminescent aggregates in aqueous solution [30–34]. So, these tetrapyrrole molecules seem to be the best candidates for researching the influence of aggregation on the FRET efficiency in the QD–molecule complexes.

In this work, we investigate nonconjugated complexes of sulfonated hydroxyaluminium phthalocyanines (PcS*_z_*) molecules with CdSe/ZnS QDs in an aqueous solution. Previously [27], we found that a reduction in the intracomplex FRET and phthalocyanine molecule PL quantum yield was observed with increase in the PcS*_z_* concentration in the mixture. We show in this paper that aggregation of PcS*_z_* molecules leads to a concentration dependence of the photophysical properties of the complexes. For the first time this concentration dependence has been well described by the developed model taking into account the heterogeneity of QD–monomer and QD–aggregate complexes. We show that a reduction in the concentration of phthalocyanine aggregates in complex with QDs results in a significant increase in efficiency of FRET between QDs and monomeric molecules.

## Results and Discussion

### QD–phthalocyanine complex formation

**Figure 1:**
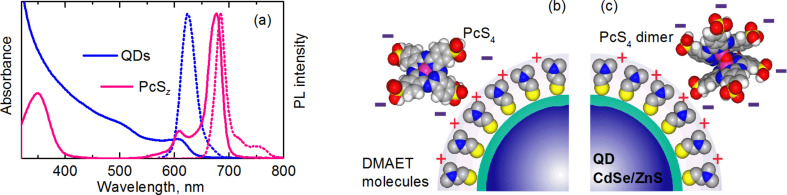
(a) UV–vis absorption (solid lines) and normalized PL spectra (dotted lines) of DMAET capped CdSe/ZnS quantum dots and PcS*_z_* in aqueous solution. Schemes of linkage of DMAET-capped CdSe/ZnS QD and sulfonated Al(OH)phthalocyanine complexes in aqueous solution by the example of PcS_4_: (b) QD–PcS_4_ monomer complex, (c) QD–PcS_4_ dimer complex.

Water soluble CdSe/ZnS quantum dots capped with 2-(dimethylamino)ethanethiol (DMAET) with a core diameter of 5 nm [35] were used in the study. UV–vis absorption and PL spectra of free CdSe/ZnS quantum dots and PcS*_z_* molecules are presented in Figure 1a. As seen in Figure 1a, a high spectral overlap between QD PL and PcS*_z_* absorption allows FRET conditions to be satisfied.

In aqueous solution, the sulfo groups PcS*_z_* dissociate at a neutral pH and acquire a negative charge. Therefore, mixing of aqueous solutions of DMAET QDs and PcS*_z_* leads to the formation of QD–DMAET–PcS*_z_* complexes as a result of the electrostatic interaction between phthalocyanine and positive DMAET solubilizer molecules on the surface of the QDs. Figure 1b shows the scheme of the linkage of PcS*_z_* to DMAET QDs by the example of PcS_4_.

Previously [27] we investigated luminescent properties of QD and PcS*_z_* mixtures with different acceptor/donor ratio. Nonluminescent PcS*_z_* aggregates were formed in complexes, as schematically shown in Figure 1c. This led to an exponential drop of the quantum yield of PcS*_z_* and of the efficiency of energy transfer in complexes with an increasing number of PcS*_z_* molecules per quantum dot. It is evident that increasing the concentration of PcS*_z_* in our samples lead to a decrease of PL of PcS*_z_* because of nonluminescent PcS*_z_* aggregates in the complexes. At the same time a thorough analysis is needed to find a correlation between the FRET efficiency and the probability of the formation of PcS*_z_* aggregates.

The FRET efficiency of a complex of a quantum dot with *m* independent acceptors arrayed around its center at a fixed distance *R* can be determined as follows [15,36]:

[1]
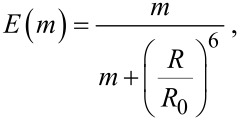


where *R*_0_ is the critical radius, i.e., the separation distance between donor and acceptor at which the FRET probability is equal to the probability of a spontaneous deactivation of the excited donor state. It can be expressed as follows:

[2]
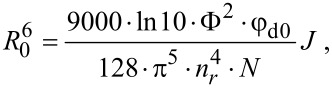


where φ_d0_ is the QY of PL of the energy donor in the absence of an acceptor; Φ^2^ is the orientation factor; *n_r_* is the refractive index; *N* is Avogadro’s number and *J* is the overlap integral:

[3]
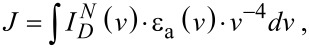


where 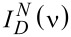 is the normalized PL spectrum of the donor (
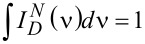
); ε_a_(ν) is the extinction of the acceptor; and ν is the wavenumber.

In the QD–DMAET–PcS*_z_* complex multiple DMAET solubilizer molecules are attached to the surface of each QD. The number of DMAET binding sites on the QD surface approximately 140 for the CdSe/ZnS quantum dots with a core diameter of 5 nm [37]. As a consequence, addition of the PcS*_z_* molecules to the mixture results in a Poisson distribution of molecules bound to the QDs [17]:

[4]
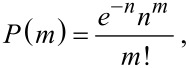


where *P*(*m*) is the probability of a QD having *m* acceptor molecules, *n* is the concentration ratio n = *C*_a_/*C*_d_, *C*_a_ and *C*_d_ are the concentrations of PcS*_z_* acceptor and QD donor in the mixture, respectively.

To estimate ensemble-average observed FRET efficiency we used an approach that is quite similar to that of Beane and co-workers [17]. In that work the ensemble-average quenching efficiency of QDs (i.e., the equation that takes into account all bonded quantum dots in the mixture) was determined as follows:

[5]
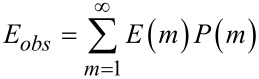


However, we prefer to use an equation for the ensemble-average FRET efficiency from one quantum dot to *m* PcS*_z_* acceptors, i.e., the ensemble-average FRET efficiency in one complex for each fixed *n*. For this, we simply normalize Equation 5 to the fraction of bonded quantum dots in the mixture:

[6]
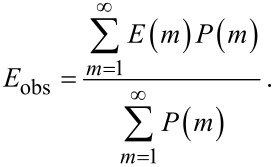


Here, 
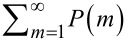
 is the fraction of quantum dots bound in a complex with the acceptor. Experimentally the FRET efficiency *E* can be determined using values of donor PL quenching and acceptor PL enhancement. However, in complexes with competitive sources of donor quenching only the approach based on the analysis of experimental data on the PL intensity of sensitized acceptor molecules is applicable. The possible change in QY of the acceptor PL should be also taken into account. For this, we estimate a relative QY of the acceptor PL in complexes with QDs under direct photoexcitation 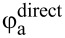 using a comparative method [38–39]:

[7]
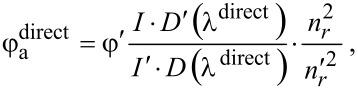


where φ′ is the PL QY of the reference fluorophore, *I* is the integrated PL intensity, *D* is the optical density at the wavelength of the direct PL excitation of the acceptor in complexes, λ^direct^, (no FRET from QD to the molecules is available), and *n* is the refractive index of the solvent. The apostrophe denotes the respective values of the reference fluorophore.

With this assumptions in place, we estimate the FRET efficiency from one quantum dot to multiple acceptor molecules in the mixture from the ratio between the QY of sensitized (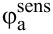) and directly excited (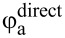) PL of the acceptors bound to donors [38]:

[8]
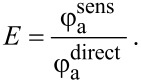


The quantities used in Equation 8, directly determined from the experimental data, are discussed in detail in the Supporting Information File 1. Equation 8 allows for the determination of the average FRET efficiency of one complex in the mixture for each fixed *n.*

**Figure 2:**
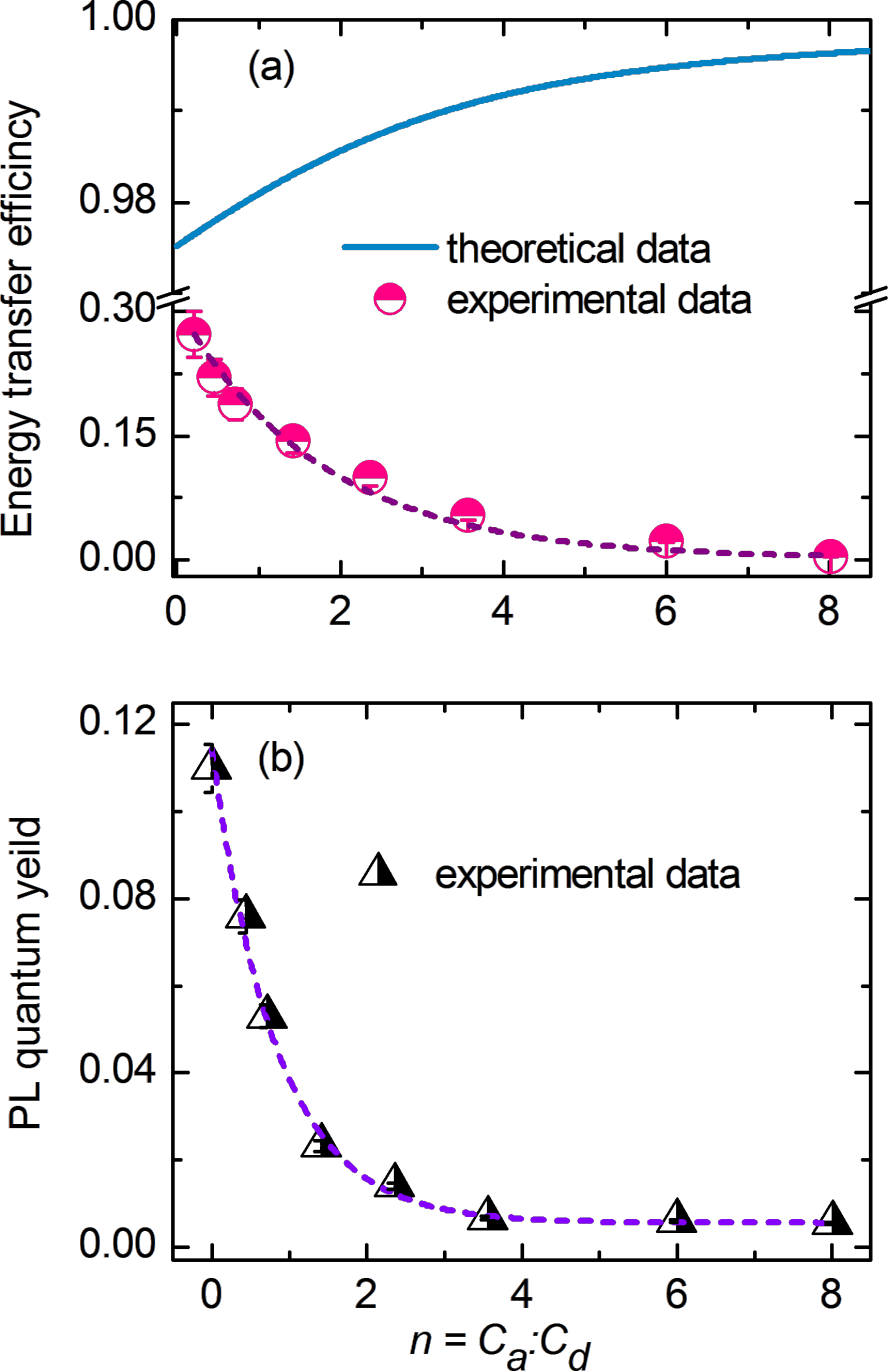
(a) Energy transfer efficiency calculated from experimental and theoretical data using Equation 8 and Equation 6, respectively. (b) QY of directly excited PL of the PcS*_z_* molecules calculated using Equation 8 in QD/PcS*_z_* complexes as a function of the relative acceptor concentration *n*. Dashed lines are to guide the eye.

In Figure 2a, we plot the FRET efficiency calculated from experimental data using Equation 8 and the theoretically possible FRET efficiency in a QD/PcS*_z_* complex calculated using Equation 6 with the critical radius *R*_0_ = 5.9 nm and the distance between the QDs and PcS*_z_* being *R* = 3.5 nm. This distance was considered as the sum of the QD radius (2.5 nm), ZnS shell thickness (0.4 nm) and length of DMAET molecules (0.6 nm). For this distance, the maximum of theoretical FRET efficiency of approximately 100% even for *n* < 1 was predicted using Equation 6, please see blue curve in Figure 2a.

As clearly seen from Figure 2a, at the lowest PcS*_z_* concentration (*n* = 0.1), the experimental intracomplex FRET efficiency is equal to 30%, which is about a third of the theoretically predicted value. The low FRET efficiency in complexes of QDs and monomeric phthalocyanine molecules is commonly observed in experimental studies [40] and can be explained by the formation of a new non-radiative channels of dissipation of the photoexcitation energy with rate constants higher than that of FRET. PL quantum yield of the PcS*_z_* molecules in complexes with *n* = 0.1, calculated according to Equation 7, is 12% (as seen in Figure 2b). This is practically the same as for free molecules in aqueous solution [41]. An increase of *n* leads to a sharp exponential drop of QY of the PcS*_z_* molecules, as well of the FRET efficiency instead of a rise as expected from the theoretical curve.

**Figure 3:**
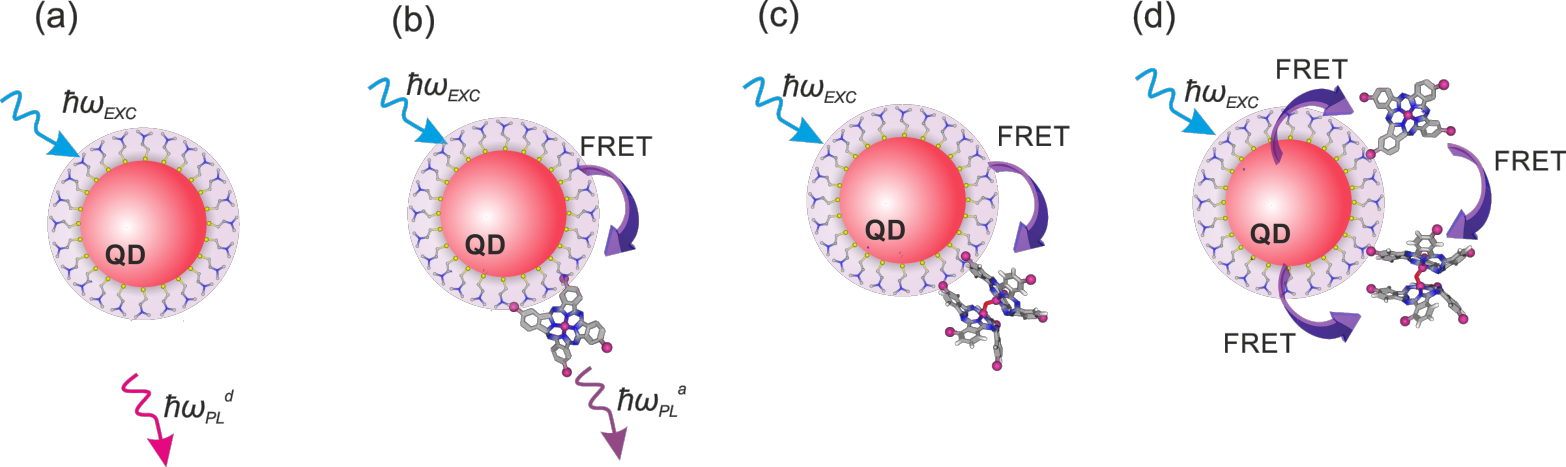
Heterogeneous system of QD–molecule complexes, which consists of free QDs (a), and complexes of QD with molecule monomers (b) and aggregates (c) as well as with both monomers and aggregates (d).

To explain how the formation of non-luminescent acceptor aggregates leads to an exponential decrease of the observed FRET efficiency we proposed the following model describing the heterogeneous system of QD/molecule complex:

1. The heterogeneous system consists of free QDs and complexes of types (a), (b) and (c), which are presented in Figure 3. The number of molecules bound in a complex per QD obeys a Poisson distribution. All molecules are bound in complexes with QDs, but free QDs can be present in the mixture.
2. Molecules in the complexes can be present as monomers (case (a) in Figure 3) or as non-luminescent aggregates formed by adjoining molecules bound to the QD (cases (b) and (c) in Figure 3).
3. The probability of aggregate formation increases with increasing relative molecule concentration *n* in the mixture, resulting in a concentration dependence of the photophysical properties of the complexes.
4. QDs are energy donors and molecules are energy acceptors during energy transfer within complexes. QD photoluminescence is completely quenched in complexes with acceptors. The PL quantum yield of monomeric acceptors in the complexes is constant.
5. Molecular aggregates, if present, act as energy acceptors. Here, QDs and monomeric molecules act as energy donors.

We propose that complexes containing at least one nonluminescent aggregate do not luminesce, i.e., both the QD PL and the PL of the monomers are completely quenched. In order to describe the concentration dependence of the probability of the formation of complexes with aggregates, we introduce the parameter α, the maximum number of molecules in complex with QDs without the formation of molecular aggregates.

In heterogeneous systems of QD–molecule complexes, where molecules can exist as monomers or aggregates, the total concentration of the QDs (donors), *C*_d,h_, and molecules (acceptors), *C*_a,h_ can be described by:

[9]
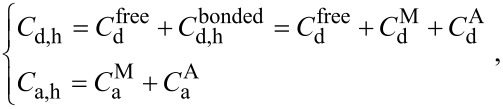


where 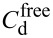 is the concentration of free donors in solution and 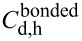 is the concentration of donors bonded in complexes with acceptors. 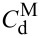 and 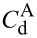 are the concentrations of donors bound to acceptor monomers and aggregates, respectively. 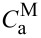 and 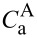 are the concentrations of acceptor in monomeric and aggregated forms in complexes, respectively.

Typically, the absorption spectra of the acceptor aggregates differ from those of the monomeric form. Therefore, Equation 9 can be transformed using the Lambert–Beer law into:

[10]
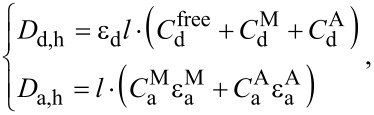


where 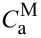, 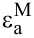 and 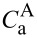, 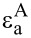 are the concentrations and extinction coefficients of acceptor molecules in monomeric and aggregate forms, respectively. 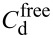 and 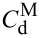, 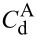 are the concentrations of unbound donors, and of donors bound with monomers and aggregates respectively. ε_d_ is the extinction coefficient of donor and *l* is the path length.

In the framework of the proposed model the probability of PcS*_z_* aggregation in the complexes is a function of *n* and depends on model parameter α. Therefore, 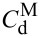, 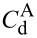, 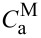 and 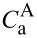 are also functions of *n* and α. A detailed derivation of these functions 
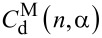
, 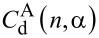, 
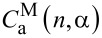
 and 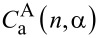 is presented in Supporting Information File 2.

**Figure 4:**
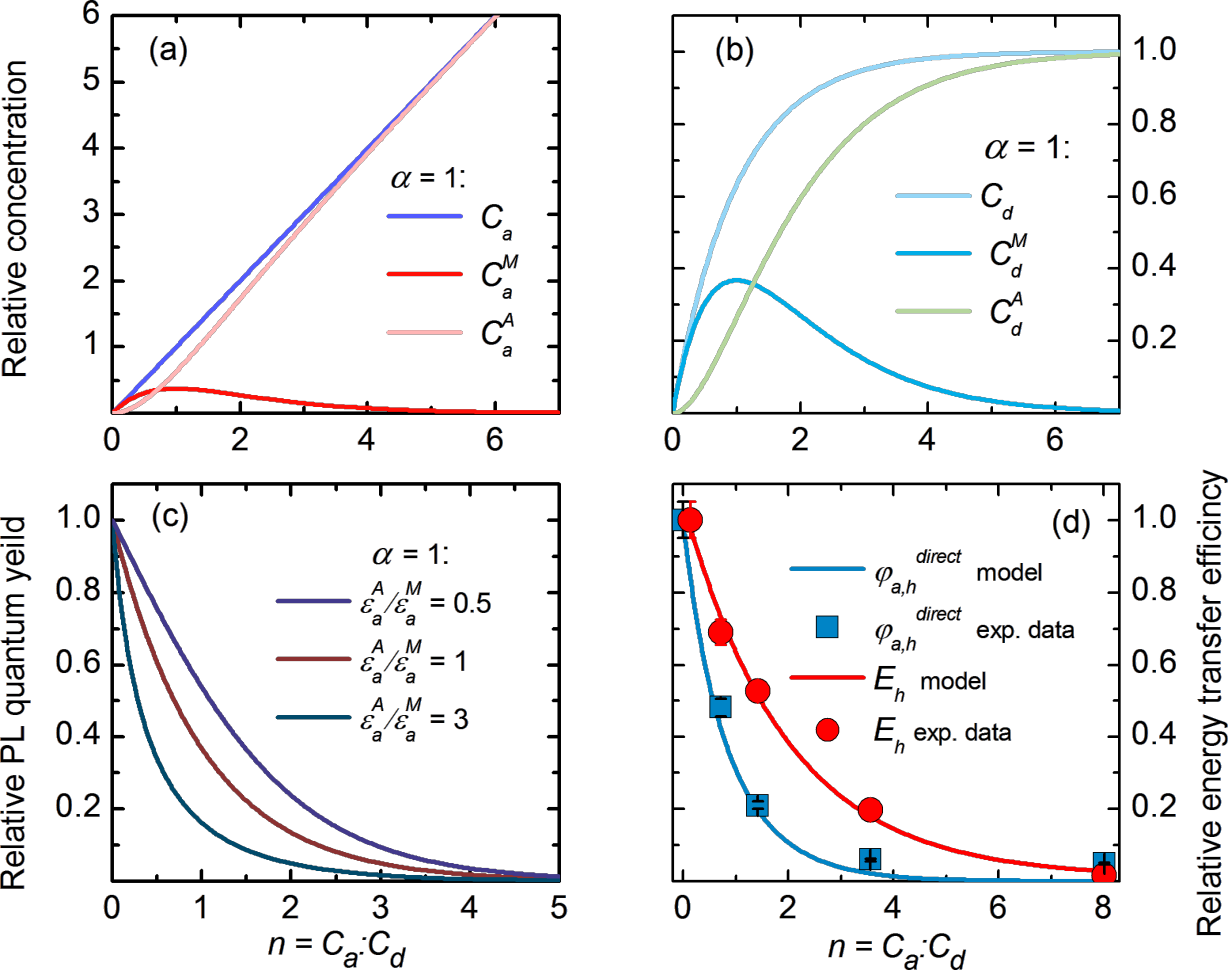
Typical results for a heterogeneous system of QD–molecule complexes, calculated from the model with α = 1. Relative concentrations of acceptor molecules (a) and QD donors (b) in complexes and QD–monomer/QD–aggregate complexes as a function of the relative acceptor concentration *n*. (c) Dependencies of relative acceptor PL QY (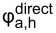) on relative acceptor concentration *n* for different ratios of monomer and aggregate extinction coefficients. (d) Experimental data of the normalized energy transfer efficiency (red spheres) and normalized acceptor PL QY (blue squares) in a heterogeneous system of QD–molecule complexes as a function of *n* calculated with Equation 7 and Equation 8, respectively. Continuous lines are the fitting of experimental data with *E*_h_ (red line) and 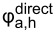 (blue line) curves calculated from the model described above with Equation 11 and Equation 12 (α = 1 and extinction ratio 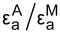 = 1.20.

Figure 4a,b shows the calculated dependencies for the “worst case scenario” when only complexes with one molecule per QD do not have acceptor aggregates and, therefore, can luminesce (i.e., α = 1). For the chosen model parameter, as it is clearly seen from Figure 4a, the concentration of acceptor molecules in aggregates, 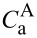, rapidly increases with *n*, and at *n* = 0.73 the number of acceptor molecules in aggregates is equal to the number of acceptor molecules in monomeric form. Figure 4b demonstrates that at equimolar concentrations of QDs and molecules approximately 30% of the complexes contain nonluminescent aggregates and do not luminesce. It can clearly be seen that the concentration of nonluminescent complexes, similar to the concentration of acceptor aggregates, increases rapidly with increase of *n*.

In a heterogeneous system of complexes the QY of directly excited acceptor PL (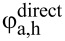) has the physical meaning of the average PL QY. Obviously, it depends on *n* and α since the probability of acceptor aggregate formation is a function of *n* and α. The obtained expression for the ensemble-averaged quantum yield of directly excited PL of acceptor molecules in heterogeneous system explains the observed concentration-dependent exponential decrease of acceptor PL quantum yield:

[12]
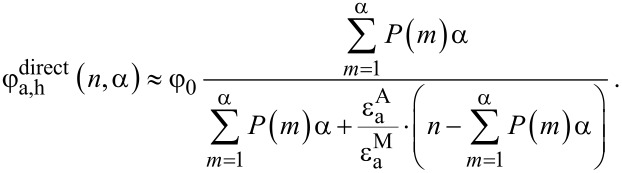


Details of these calculations are given in the Supporting Information File 3. As expected, the extinction ratio 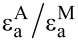 at the wavelength of direct acceptor photoexcitation significantly affects the slope of the 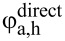 curve. The dependencies of 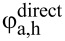 on *n* for a number of different ratios 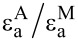 at α = 1 are shown in Figure 4c.

Equation 8 applies to homogeneous systems in which the acceptors are monomers and underestimates the energy transfer efficiency when applied to the formation of nonluminescent complexes with molecular aggregates. Since the optical densities of donor and acceptor in QD–monomer and QD–aggregate complexes cannot be measured separately, a mean energy transfer efficiency in heterogeneous systems of complexes, *E*_h_(*n*,α), can be only calculated from experimental data. An analytical expression that describes the mean ensemble-average energy transfer efficiency in heterogeneous systems of complexes based on the proposed model (for details see Supporting Information File 3) is as follows:

[11]
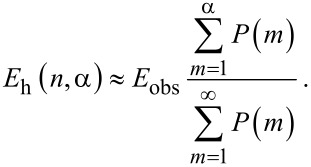


Equation 11 clearly demonstrates that in heterogeneous systems of complexes with high probability of aggregates formation (i.e., with low α *v*alues) an increase of the acceptor concentration will result in an exponential decrease of ensemble-average energy transfer efficiency. So, the proposed model demonstrates that an increase in the probability of acceptor aggregation in complexes with QDs leads to an exponential decrease of *E*_h_ and 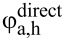 as functions of *n* and α.

In accordance with the proposed model, concentration dependencies of two independently determined parameters –energy transfer efficiency and the QY of acceptor PL, which are presented in Figure 2– were approximated by corresponding simulated curves. Fitting of two independent experimental curves by model functions that include the parameter α allows one to qualitatively evaluate the distribution of the acceptor in complexes QD–monomer and QD–aggregate at different *n*.

The normalized experimental energy transfer efficiency and the quantum yield of directly excited acceptor PL (red and blue dots, respectively) fitted by the simulated curves *E*_h_(*n*,α) and 
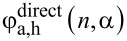
 (red and blue solid lines, respectively) are presented in Figure 4d. For fitting of both experimental curves a value of α = 1 was used that indicates that aggregation of PcS*_z_* occurs in all instances when more than one PcS*_z_* molecule is bound in a complex with a QD. It should be noted that for α = 1, the best approximation of the dependence of the QY for directly excited acceptor PL on *n* was obtained with a value of the extinction ratio 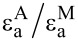 = 1.20. This is in good accordance with the extinction ratio 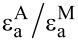 = 1.15 ± 0.05 obtained from the absorption spectra. The relevant calculations of the extinction of PcS*_z_* molecules in aggregates are presented in the Supporting Information File 4.

The most favorable condition for phthalocyanine molecule aggregation in an aqueous solution is a complete or partial neutralization of the molecular charges of phthalocyanine [33]. This can occur upon their binding to the positively charged stabilizer molecules on the QD surface. It is expected that phthalocyanine molecules with two sulfonic groups will primarily form nonluminescent aggregates in the complexes. Then, a decrease in the percentage of such phthalocyanine molecules in the mixture should lead to a decrease in the probability of phthalocyanine molecule aggregation in complex with QDs.

Paper chromatography revealed two chromatogram regions enriched in tetrasulfonated hydroxyaluminium phthalocyanine PcS_4_ and in disulfonated hydroxyaluminium phthalocyanine PcS_2_. Both the PcS_2_ and PcS_4_ in solution with QDs demonstrated the formation of complexes, with energy transfer quenching the QD PL and enhancing the Pc PL. Our estimations using Equation 8 have shown that the experimental FRET efficiency is equal to (35 ± 10)% for all samples at low phthalocyanine concentrations.

**Figure 5:**
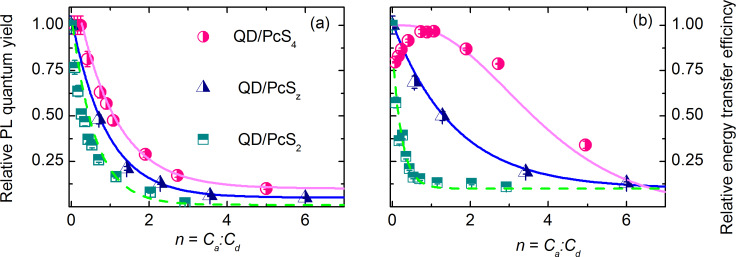
Dependencies of the relative QY of directly excited acceptor PL (a) and of the energy transfer efficiency (b) in complexes of QD and phthalocyanines with different numbers of sulfo groups on the relative phthalocyanines concentration *n* in the mixtures: PcS_4_ (red circles), PcS_2_ (green squares), original PcS_z_ mixture (black triangles). Symbols in (a) and (b) are experimental data of QY of directly excited acceptor PL and energy transfer efficiencies, calculated using Equation 7 and Equation 8, respectively, and fitted with 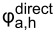 and *E*_h_ curves (solid lines), calculated from the model (Equation 12 and Equation 11) with values of: α = 3 and 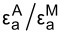 = 3 for PcS_4_ (red line); α = 1 and 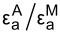 = 1.2 for PcS_z_ (black line); α = 1 and 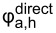 = 3 for PcS_2_ (green line). The blue line is to guide the eye.

Figure 5a,b shows normalized values of the acceptor QY and of the energy transfer efficiency calculated from experimental data using Equation 7 and Equation 8, respectively, for complexes of QDs with PcS_4_ and PcS_2_ (red circles and blue squares, respectively). Similar dependencies for QD complexes with the original PcS*_z_* mixture (Photosens^®^) are shown for comparison.

As clearly seen from Figure 5 enriching the mixture of QDs with PcS_4_ or PcS_2_ leads to significant changes in the experimental dependencies of the QY of phthalocyanine PL and of the energy transfer efficiency on *n*. In the mixture enriched with PcS_4_ a reduction of QY of phthalocyanine PL begins at *n* ≈ 0.3 in contrast with original PcS*_z_* mixture (*n* ≈ 0.1). Importantly, the energy transfer efficiency in the case of PcS_4_ demonstrates the opposite dependence on *n* when compared to that for the original mixture. Numerical simulations show that the experimental dependencies of QY of phthalocyanine PL on *n* are well fitted with a 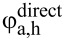 curve calculated using Equation 12 from the model with α = 3. The experimental energy transfer efficiency for the PcS_4_ fraction was also well fitted with a *E*_h_ curve, calculated from the model with α = 3 (see Figure 5b). These results are in qualitative agreement with the model, indicating the crucial role of acceptor aggregates on the photophysical properties of the QD–PcS*_z_* complexes.

As expected, in the mixture enriched with PcS_2_ the QY of phtalocyanine PL and the energy transfer efficiency exhibit a more pronounced decrease with *n* compared to the original PcS*_z_* mixture. Fitting of the experimental data with 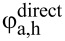 curve gives values of α = 1 and 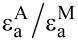 = 3. However, the energy transfer efficiency for the PcS_2_ fraction was found to decrease with *n* more rapidly than the model predicts (the blue curve in Figure 5b is to guide the eye). It is possible that complexes with acceptor aggregates may not obey Poisson statistics because probabilities of binding a free PcS*_z_* molecule with QD solubilizer and with PcS*_z_* molecules bound with QDs are not the same. The proposed model, which demonstrates *n*-dependence of energy transfer efficiency due to aggregation of molecules in the complexes with QDs, was simplified and does not take into account all the experimental conditions. In particular, the model assumes a fulfillment of Poisson distribution of molecules in the complexes. We are going to develop the model to take into account non-Poisson statistics in the QD–molecules complexes in the next stage.

## Conclusion

The nonradiative intracomplex energy transfer in nonconjugated complexes of sulfonated phthalocyanines (PcS*_z_*) molecules with CdSe/ZnS QDs in an aqueous solution has been studied by absorption and PL spectroscopy. A sharp decrease of the energy transfer efficiency with increasing PcS*_z_* (acceptor) concentration has been found. This effect has been explained by the formation of nonluminescent aggregates of PcS*_z_* in the complexes with CdSe/ZnS QDs. A corresponding model of the aggregate formation with growth of relative concentration *n* of the PcS_z_ has been developed. The model demonstrates that aggregation of molecules results in a dependence on *n* of the photophysical properties of the complexes, including reduction of the energy transfer efficiency with increasing *n*. Experimental data on the QY of phthalocyanine PL and FRET efficiency are in good agreement with the proposed model. We demonstrate the possibility to increase efficiency of FRET between QDs and monomeric molecules by a reduction in the concentration of phthalocyanine aggregates in complex with QDs. We believe that the model will help to better understand photophysical processes in QD–molecule complexes for design of systems with the desired spatial arrangement of QD–molecule complexes for PDT.

## Experimental

### Materials

The photosensitizer Photosens^®^ was obtained from NIOPIK (Russia). At present, the Photosens^®^ is used clinically for PDT [42]. Photosens^®^ is a mixture of sulfonated hydroxyaluminium phthalocyanines (PcS*_z_*) with different numbers of sulfo groups per molecule, with *z* = 2, 3 or 4. So, in an aqueous solution PcS_z_ is a mixture of phthalocyanine molecules with a different number of negative charges. Paper chromatography was used in order to obtain phthalocyanine mixtures enriched in either tetrasulfopthalocyanine (PcS_4_) or disulfopthalocyanine (PcS_2_) [43]. Toluene, methanol, trioctylphosphine oxide (TOPO) and 2-(dimethylamino)ethanethiol (DMAET) were purchased from Aldrich.

### Quantum dot synthesis

CdSe/ZnS quantum dots with 5 nm cores were synthesized using methods previously described in [44] In order to make the QDs water soluble, we applied a standard phase transfer procedure to the QDs, involving the replacement of trioctylphosphine oxide (TOPO) molecules on the QD surface with hydrophilic 2-(dimethylamino)ethanethiol (DMAET) molecules, producing positively charged QDs in aqueous solution.

### Complex formation

Complexes of quantum dots with phthalocyanine molecules were produced in a similar manner as described in [27] by mixing solutions of pure QDs with a concentration of *C*_d_ ≈ 1 µmol/L and PcS*_z_*. In order to study the dependencies of the QD and phthalocyanine PL intensities on the molar ratio of the components in the mixture, a concentrated solution of PcS*_z_* was sequentially added to the QD solution, creating a final mixture with a PcS*_z_* concentration in the range from 1 × 10^−8^ to 5 × 10^−6^ mol/L. All measurements were performed within two hours after preparation of the solution. UV–vis absorption and PL spectra of samples were measured for the mixture solutions after every addition step.

### UV–vis absorption and PL detection

UV–visible absorption spectra were recorded using a UV3600 (Shimadzu) spectrophotometer. Steady-state photoluminescence spectra were measured using a Cary Eclipse (Varian) spectrofluorometer. Time-resolved PL spectroscopy was performed using a time-correlated single photon counting (TCSPC) spectrometer MicroTime100, from Pico Quant, Inc. A pulsed laser operating at 405 nm with an average power of 1 mW was used for PL excitation. The pulse repetition rate was 40 MHz with pulse duration of 70 ps.

PL quantum yields of the samples were estimated by a comparative method [38–39] using Rhodamine 6G in ethanol (φ_R_ = 0.95) [45] as a reference fluorophore. Photosens^®^ has a PL QY of ca. 12% in aqueous solution [41]. The QD samples have a PL QY > 20% in hydrophobic solvents and of about 10% in aqueous solutions.

Light with wavelengths of 475 and 640 nm was used for PL excitation. At a wavelength of 475 nm, there is a local minimum of the phthalocyanine absorption and QDs can be selectively excited. For direct excitation of the phthalocyanine, a wavelength of 640 nm was chosen, since at this wavelength there is a strong *Q*(*I*) phthalocyanine absorption band, while the QD absorption is negligible. This approach allows us to easily evaluate the efficiency of energy transfer in the mixture solution and the change in the PL QY of phthalocyanine bound to QDs.

## Supporting Information

Supporting Information contains: (i) FRET efficiency estimation using acceptor PL enhancement. QD PL decay curves, (ii) derivation of the dependencies of the donor and acceptor concentrations in QD-monomer and QD-aggregate complexes on the relative acceptor concentration, (iii) derivation of analytical expressions of PL QY of the acceptor and energy transfer efficiency on *n* in a heterogeneous system, and (iv) estimation of the extinction coefficients of the PcS*_z_* molecules in monomeric and aggregated forms.

File 1 FRET efficiency estimation using acceptor PL enhancement.

File 2 Derivation of the dependencies of the donor and acceptor concentrations in QD-monomer and QD-aggregate complexes on the relative acceptor concentration.

File 3 Derivation of analytical expressions of PL QY of the acceptor and energy transfer efficiency on *n* in a heterogeneous system.

File 4 Estimation of the extinction coefficients of the PcS*_z_* molecules in monomeric and aggregated forms.
