# Supplementary material for: The influence of phthalocyanine aggregation in complexes with CdSe/ZnS quantum dots on the photophysical properties of the complexes
Source: Beilstein J Nanotechnol. 2016 Jul 13;7:1018–27. doi: 10.3762/bjnano.7.94 (PMC4979882; doi:10.3762/bjnano.7.94)
Supplement: File 1 — FRET efficiency estimation using acceptor PL enhancement. [file Beilstein_J_Nanotechnol-07-1018-s001.pdf]

Supporting Information

for

The influence of phthalocyanine aggregation in complexes with CdSe/ZnS quantum dots on the photophysical properties of the complexes

Irina V. Martynenko[1], Anna O. Orlova*[1], Vladimir G. Maslov[1], Anatoly V. Fedorov[1], Kevin Berwick[2] and Alexander V. Baranov[1]

Address: [1] Department of optical physics and modern natural science, ITMO University, 197101 Saint Petersburg, Russia and [2]Department of Electronic and Communications Engineering, Dublin Institute of Technology, Dublin 8, Ireland

Email: Anna Orlova* - a.o.orlova@gmail.com

* Corresponding author

FRET efficiency estimation using acceptor PL enhancement

FRET efficiency can be estimate from experimental data from the ratio between the QY of sensitized $\left(\varphi_a^{sens}\right)$ and directly excited $\left(\varphi_a^{direct}\right)$ photoluminescence of the acceptors bound to donors as follows:

$$E = \frac{\varphi_a^{sens}}{\varphi_a^{direct}} = \frac{I_a^{sens}(\lambda^d exc)}{D_d^{bonded}(\lambda^d exc)} \cdot \frac{D_a(\lambda^a exc)}{I_a^{direct}(\lambda^a exc)}, \tag{S1}$$

where $I_a^{sens}(\lambda^d exc)$, $I_a^{direct}(\lambda^a exc)$ are the acceptor-sensitized and directly excited PL intensity, respectively; $D_d^{bonded}(\lambda^d exc)$ is the optical density of donors in complexes at

the excitation wavelength of the acceptor sensitized PL; $D_a \lambda^a exc$ is the acceptor optical density at the wavelength used for direct photoexcitation of acceptors. These experimentally obtained values associated with the acceptor and donor extinction as follows:

$$\begin{cases} I_a^{sens}(\lambda^d exc) \sim I_0 \cdot \varepsilon_d^{\lambda exc} \cdot E(m) \cdot \varphi \cdot C_d^{bonded} \cdot l \\ I_a^{direct}(\lambda^a exc) \sim I_0 \cdot \varepsilon_a^{\lambda exc} \cdot \varphi \cdot C_a \cdot l \\ D_d^{bonded}(\lambda^d exc) = \varepsilon_d^{\lambda exc} \cdot C_d^{bonded} \cdot l \\ D_a(\lambda^a exc) = \varepsilon_a^{\lambda exc} \cdot C_a \cdot l \end{cases}, \qquad \text{(S2)}$$

where I_0 is the intensity of the exciting radiation; $\varepsilon_d^{\lambda exc}, \varepsilon_a^{\lambda exc}$ are donor and acceptor extinction coefficients at the photoexcitation wavelength, $E(m)$ is FRET efficiency in complex of quantum dot with *m* independent acceptors, φ is acceptor PL quantum yield; *l* is the path length. C_a is the acceptor concentration in the mixture, C_d^{bonded} is the concentration of donors that are bound in complex with acceptor in the mixture.

Combining Equation S2 with Equation S1 gives:

$$E \equiv E(m) \qquad \text{(S3)}$$

So, Equation S1 allows one to determine the average FRET efficiency in one complex in the mixture for every fixed *n.*

However, this expression can be used only in case when optical density of bonded donors D_d^{bonded} can be determined. In our case, optical density of bonded quantum dots cannot be measured experimentally, so we use following approach. D_d^{bonded} correlates with the concentration of donors (C_d^{bonded}) that are bonded in complexes with acceptors as:

$$C_d^{bonded} = C_d \cdot f \qquad \text{(S4)}$$

where C_d is the total concentration of donors and *f* is the fraction of donors bonded in complexes with acceptor molecules. Assuming only free donors luminesce in the mixture solution, *f* can be experimentally estimated as $f = 1 - (I/I_0)$, where *I* and I_0 are donor PL intensities in the presence/absence of the acceptor.

In the QD/PcS_z complexes the PL of quantum dots was completely quenched because QD PL lifetime decays in QD-PcS_z mixtures were equal to these of free QDs in aqueous solution. PL decay curves for free DMAET-QDs in aqueous solution and in complexes with PcS_z (with relative acceptor concentration in complexes $n \approx 1.5$) are presented in

Figure S1. As seen from Figure S1, QD PL decay remained unchanged in complexes with phthalocyanine. The same results were obtained for all complexes in work (data not shown). Increased signal to noise ratio in PL decay curve of QD in complexes in Figure S1 is attributed to pronounce quenching of QD photoluminescence in complexes.

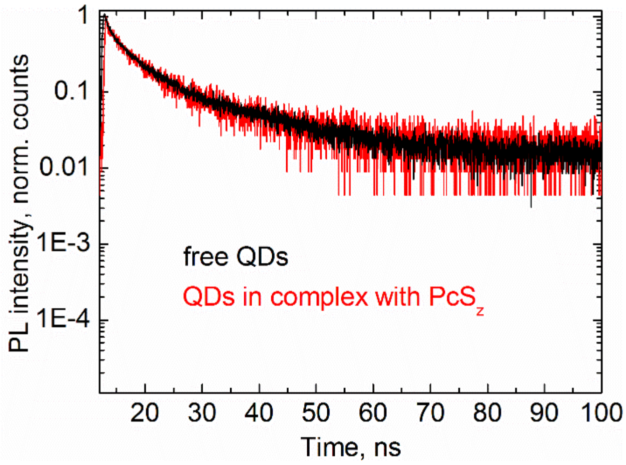

Figure S1: PL decay curves for free DMAET-QDs in aqueous solution and in complexes with PcS_z (relative acceptor concentration in complexes $n \approx 1.5$).

So, using Equation S1 allows us to determine average FRET efficiency in one QD/PcS_z complex in the mixture for every fixed n.
