# Supplementary material for: The influence of phthalocyanine aggregation in complexes with CdSe/ZnS quantum dots on the photophysical properties of the complexes
Source: Beilstein J Nanotechnol. 2016 Jul 13;7:1018–27. doi: 10.3762/bjnano.7.94 (PMC4979882; doi:10.3762/bjnano.7.94)
Supplement: File 2 — Derivation of the dependencies of the donor and acceptor concentrations in QD-monomer and QD-aggregate complexes on the relative acceptor concentration. [file Beilstein_J_Nanotechnol-07-1018-s002.pdf]

Supporting Information

for

The influence of phthalocyanine aggregation in complexes with CdSe/ZnS quantum dots on the photophysical properties of the complexes

Irina V. Martynenko[1], Anna O. Orlova*[1], Vladimir G. Maslov[1], Anatoly V. Fedorov[1], Kevin Berwick[2] and Alexander V. Baranov[1]

Address: [1] Department of optical physics and modern natural science, ITMO University, 197101 Saint Petersburg, Russia and [2]Department of Electronic and Communications Engineering, Dublin Institute of Technology, Dublin 8, Ireland

Email: Anna Orlova* - a.o.orlova@gmail.com

* Corresponding author

Derivation of the dependencies of the donor and acceptor concentrations in QD-monomer and QD-aggregate complexes on the relative acceptor concentration

The dependence of the concentration of donors bonded with acceptors, C_d^{bonded} on the relative acceptor concentration in the mixture, $n = C_a / C_d$, can be easily calculated from the Poisson distribution:

$$P(m) = \frac{n^m}{m!} e^{-n} . \qquad \text{(S1)}$$

The fraction of bonded donors is defined as:

$$\frac{C_d^{bonded}}{C_d} = \sum_{m=1}^{\infty} P(m) \quad \text{(S2)}$$

and the concentration of bonded donors is:

$$C_d^{bonded}(n) = C_d \sum_{m=1}^{\infty} P(m) \quad \text{(S3)}$$

Since the parameter α is the maximum number of acceptor molecules in the complex, where there is no formation of acceptor aggregates, the concentration of donors bonded with acceptor monomers can be defined as a sum of the concentrations of donors bonded with 1,...,α acceptor molecules:

$$C_d^M(n,\alpha) = C_d \cdot \sum_{m=1}^{\alpha} P(m). \quad \text{(S4)}$$

We can express the dependencies of the concentration of acceptor molecules in both the aggregated and the monomeric forms on the QD surface on n. Since we assume that the acceptor in monomeric form exists only in complexes, where the maximum number of molecules on the QD surface is equal to α, the monomer concentration is equal to the sum of the concentration of QD donors bonded with acceptor monomers multiplied by 1,...,α, which can be obtained from Equation S4:

$$C_a^M(n,\alpha) = C_d \cdot \sum_{m=1}^{\alpha} P(m)\alpha\,. \quad \text{(S5)}$$

The concentration of the aggregated acceptor molecules can be obtained by subtracting the monomer concentration (Equation S5) from the total acceptor concentration:

$$C_a^A(n,\alpha) = C_a - C_a^M(n,\alpha) = C_a - C_d \cdot \sum_{m=1}^{\alpha} P(m)\alpha \quad \text{(S6)}$$
