# Supplementary material for: The influence of phthalocyanine aggregation in complexes with CdSe/ZnS quantum dots on the photophysical properties of the complexes
Source: Beilstein J Nanotechnol. 2016 Jul 13;7:1018–27. doi: 10.3762/bjnano.7.94 (PMC4979882; doi:10.3762/bjnano.7.94)
Supplement: File 3 — Derivation of analytical expressions of PL QY of the acceptor and energy transfer efficiency on n in a heterogeneous system. [file Beilstein_J_Nanotechnol-07-1018-s003.pdf]

Supporting Information

for

The influence of phthalocyanine aggregation in complexes with CdSe/ZnS quantum dots on the photophysical properties of the complexes

Irina V. Martynenko[1], Anna O. Orlova*[1], Vladimir G. Maslov[1], Anatoly V. Fedorov[1], Kevin Berwick[2] and Alexander V. Baranov[1]

Address: [1] Department of optical physics and modern natural science, ITMO University, 197101 Saint Petersburg, Russia and [2]Department of Electronic and Communications Engineering, Dublin Institute of Technology, Dublin 8, Ireland

Email: Anna Orlova* - a.o.orlova@gmail.com

* Corresponding author

Derivation of analytical expressions of PL QY of the acceptor and energy transfer efficiency on *n* in a heterogeneous system

In the case of a heterogeneous system, we can only measure the total optical density for the acceptors (both monomers and aggregates) as well as just total optical density of bonded donors (i.e. donors bonded with both acceptor aggregates and monomers). So in a heterogeneous system of complexes ensemble-averaged QY of directly excited acceptor PL ($\varphi_{a,h}^{direct}$) and energy transfer efficiency (E_h) are defined as:

$$\varphi_{a,h}^{direct} = \frac{I_a^{direct}}{D_a^M + D_a^A} \qquad \text{(S1)}$$

$$E_h = \frac{I_{a,h}^{sens}}{D_d^M + D_d^A} \cdot \frac{D_a^M + D_a^A}{I_{a,h}^{direct}} \qquad (S2)$$

Where $I_{a,h}^{direct}$ and $I_{a,h}^{sens}$ are the directly excited and sensitized acceptor PL, respectively, where D_a^M and D_a^A are the optical densities of acceptor molecules in monomeric and aggregate forms in complexes, respectively; D_d^M, D_d^A are the optical densities of donors bound with monomers and aggregates respectively.

Optical densities of donors and acceptors in heterogeneous system are simply derived as:

$$\begin{aligned} D_a^M &= C_a^M \varepsilon_a^M l \\ D_a^A &= C_a^A \varepsilon_a^A l \\ D_d^M &= C_d^M \varepsilon_d l \\ D_d^A &= C_d^A \varepsilon_d l \end{aligned} \qquad (S3)$$

Where C_a^M, ε_a^M and C_a^A, ε_a^A are the concentrations and extinction coefficients of acceptor molecules in monomeric and aggregate forms in complexes, respectively; C_d^M, C_d^A are the concentrations donors bound with monomers and aggregates respectively; ε_d is the extinction coefficients of donor, l is the path length.

Derivation of the dependencies of the donor and acceptor concentrations in QD-monomer and QD-aggregate complexes on the relative acceptor concentration are presented in Supporting Information File 2.

The dependency of the acceptor luminescence intensity on *n* can be found by modifying the model by assuming that only acceptors in the QD/monomer complexes contribute to the measured acceptor PL in the heterogeneous system. Therefore, the intensity of the directly excited acceptor PL should be proportional to the monomer optical density at the photoexcitation wavelengths:

$$I_{a,h}^{direct}(\lambda_{exc}) \sim I_0 \cdot \varphi \cdot D_a^M = I_0 \cdot \varphi_0 \cdot \varepsilon_a^M(\lambda_{exc}) \cdot C_a^M \cdot l, \qquad (S4)$$

where I_0 is the intensity of the exciting radiation; $\varepsilon_a^M(\lambda_{exc})$ is the acceptor monomer extinction coefficient at the wavelength of PL excitation λ_{exc}; φ_0 is the PL quantum yield of monomers and C_a^M is the concentration of acceptor monomer.

Combining Equation S1 with Equation S3 and Equation S4 gives:

$$\varphi_{a,h}^{direct}(n,\alpha)\sim\frac{I_a^{direct}}{D_a^M+D_a^A}=\varphi_0\frac{\varepsilon_a^M C_a^M}{\varepsilon_a^M C_a^M+\varepsilon_a^A C_a^A}=\varphi_0\frac{\sum_{m=1}^{\alpha}P(m)\alpha}{\sum_{m=1}^{\alpha}P(m)\alpha+\frac{\varepsilon_a^A}{\varepsilon_a^M}\left(n-\sum_{m=1}^{\alpha}P(m)\alpha\right)} \quad \text{(S5)}$$

For a heterogeneous system of complexes, the intensity of the sensitized acceptor PL depends on the concentration of donors bound to monomers. It is also necessary to take into account the variation of the $\varphi_{a,h}^{direct}$ value with n accordingly to Equation S5:

$$I_{a,h}^{sens}(\lambda_{exc})\sim I_0\cdot\varepsilon_d(\lambda_{exc})\cdot E(m)\cdot\varphi_{a,h}^{direct}\cdot C_d^M\cdot l, \quad \text{(S6)}$$

where $\varepsilon_d(\lambda_{exc})$ is the donor extinction coefficient at the wavelength of PL excitation λ_{exc}; $E(m)$ is FRET efficiency in complex of quantum dot donor with m independent acceptor monomers.

Combining Equation S2 with Equation S3 and Equation S5 gives:

$$E_h(n,\alpha)=\frac{I_{a,h}^{sens}}{D_d^M+D_d^A}\cdot\frac{D_a^M+D_a^A}{I_{a,h}^{direct}}\sim E_{obs}\frac{C_d^M}{C_d^{bonded}}=E_{obs}\frac{\sum_{m=1}^{\alpha}P(m)}{\sum_{m=1}^{\infty}P(m)} \quad \text{(S7)}$$
