# Supplementary material for: The influence of phthalocyanine aggregation in complexes with CdSe/ZnS quantum dots on the photophysical properties of the complexes
Source: Beilstein J Nanotechnol. 2016 Jul 13;7:1018–27. doi: 10.3762/bjnano.7.94 (PMC4979882; doi:10.3762/bjnano.7.94)
Supplement: File 4 — Estimation of the extinction coefficients of the PcSz molecules in monomeric and aggregated forms. [file Beilstein_J_Nanotechnol-07-1018-s004.pdf]

Supporting Information

for

The influence of phthalocyanine aggregation in complexes with CdSe/ZnS quantum dots on the photophysical properties of the complexes

Irina V. Martynenko[1], Anna O. Orlova*[1], Vladimir G. Maslov[1], Anatoly V. Fedorov[1], Kevin Berwick[2] and Alexander V. Baranov[1]

Address: [1] Department of optical physics and modern natural science, ITMO University, 197101 Saint Petersburg, Russia and [2]Department of Electronic and Communications Engineering, Dublin Institute of Technology, Dublin 8, Ireland

Email: Anna Orlova* - a.o.orlova@gmail.com

* Corresponding author

Estimation of the extinction coefficients of the PcS_z molecules in monomeric and aggregated states

Figure S2 shows UV–vis absorption spectra of the PcS_z and QD solutions, which were used for the estimation of the extinction coefficients of the PcS_z molecules in monomeric and aggregated forms.

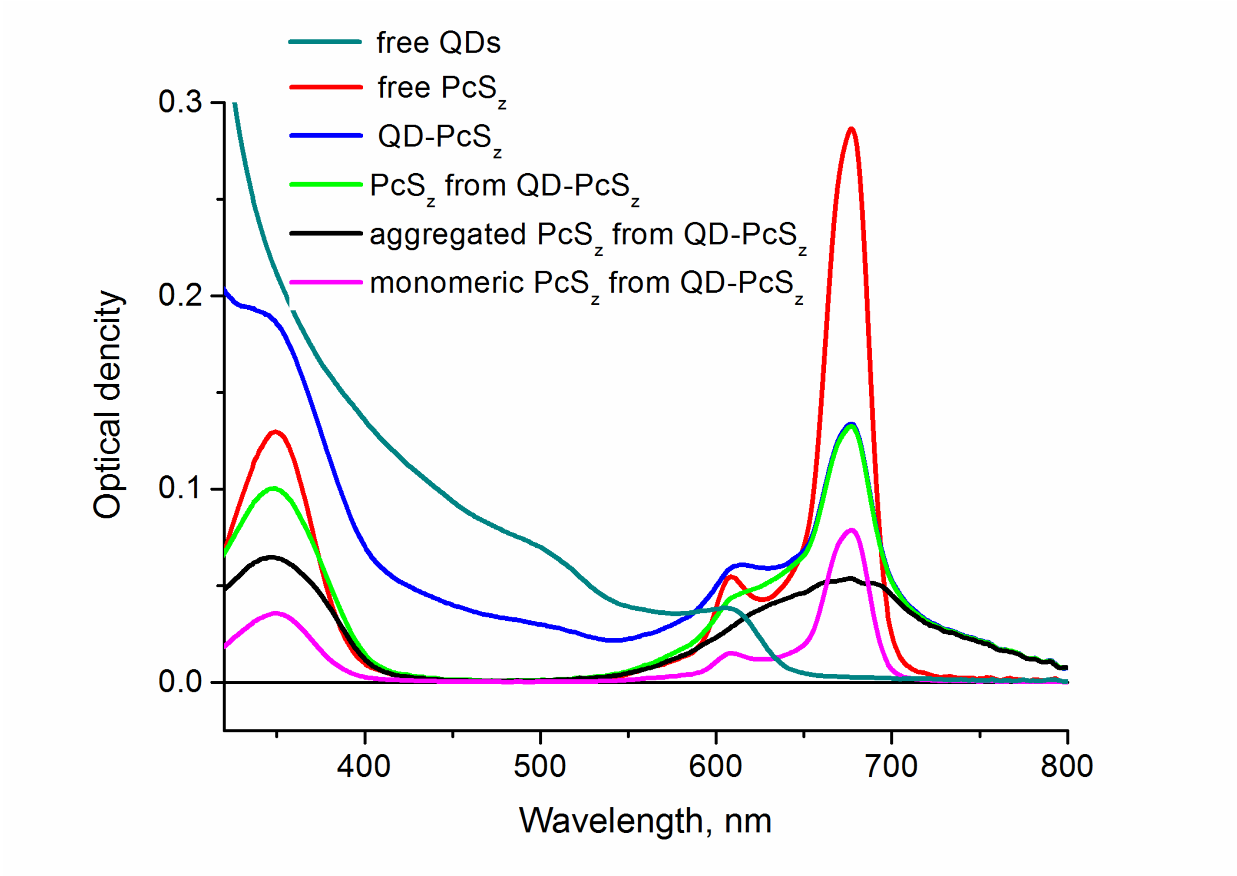

Figure S2: UV–vis absorption spectra of the PcS$_z$ and QD solutions under study.

Complexes of quantum dots with phthalocyanine molecules with a high relative concentration of PcS$_z$, n ~ 50, were produced by the addition of the stock QD solution to the free PcS$_z$ solution.

Analysis of the absorption spectrum of the mixture solution (blue curve, Figure S2) revealed PcS$_z$ aggregation in the complexes with QDs. A decrease in the intensities of the Pc Soret band (350 nm) and the Q band (675 nm), as well as the appearance of the dimer band at 644 nm, were observed.

An absorption spectrum of the PcS$_z$ in complexes with QDs (green curve) was obtained as a result of subtracting the QD absorption spectrum from the QD/PcS$_z$ mixture absorption spectrum. The absorption spectrum of aggregates (black curve, Figure S2) was derived after subtracting the contribution of the monomeric PcS$_z$ (pink curve, Figure S2) to the absorption spectrum of the PcS$_z$ in the complexes with QDs.

Using a value for the monomeric PcS$_z$ extinction coefficient of 1.75×10^5 mol^{-1}cm^2 for the Q band, the total PcS$_z$ concentration C_a and PcS$_z$ monomer concentration C_a^M were calculated from the free PcS$_z$ (red curve, Figure S2) and monomeric PcS$_z$ (pink curve, Figure S2) optical densities according to the Beer–Lambert law.

The concentration of the aggregated PcS_z molecules C_a^A was calculated from mass balance:

$$C_a^A = C_a - C_a^M . \qquad (S1)$$

The extinction coefficients of the PcS_z molecules in monomeric and aggregate forms were calculated for direct PcS_z photoexcitation at a wavelength of 640 nm.

The data obtained are listed in Table S1.

Table S1: Concentrations and extinction coefficients of monomeric and aggregated PcS_z in the complexes with QDs.

C_a, μmol/L	1.64 ± 0.08
C_a^M, μmol/L	0.45 ± 0.018
C_a^A, μmol/L	1.19 ± 0.047
$\varepsilon_a^M(640)$, mol^{-1}cm^2	(3.167 ± 0.13) × 10^4
$\varepsilon_a^A(640)$, mol^{-1}cm^2	(3.64 ± 0.14) × 10^4

The ratio of the extinction coefficients of PcS_z molecules in aggregate and monomeric forms $\varepsilon_a^A / \varepsilon_a^M$ was found to be 1.15 for PcS_z photoexcitation at a wavelength of 640 nm.
